# Interleukin-17 receptor D (Sef) is a multi-functional regulator of cell signaling

**DOI:** 10.1186/s12964-020-00695-7

**Published:** 2021-01-12

**Authors:** Shivangi Pande, Xuehui Yang, Robert Friesel

**Affiliations:** 1 Center for Molecular Medicine, Maine Medical Center Research Institute, 81 Research Drive, Scarborough, ME 04074 USA; 2 Graduate School of Biomedical Sciences and Engineering, University of Maine, Orono, ME 04496 USA

**Keywords:** Sef, Interleukin-17, Interleukin-17 receptor D (IL17RD), Fibroblast growth factor (FGF), Fibroblast growth factor receptor (FGFR), Cellular signaling

## Abstract

Interleukin-17 receptor D (IL17RD or IL-17RD) also known as Sef (similar expression to fibroblast growth factor), is a single pass transmembrane protein that is reported to regulate several signaling pathways . IL17RD was initially described as a feedback inhibitor of fibroblast growth factor (FGF) signaling during zebrafish and frog development. It was subsequently determined to regulate other receptor tyrosine kinase signaling cascades as well as several proinflammatory signaling pathways including Interleukin-17A (IL17A), Toll-like receptors (TLR) and Interleukin-1α (IL1α) in several vertebrate species including humans. This review will provide an overview of IL17RD regulation of signaling pathways and functions with emphasis on regulation of development and pathobiological conditions. We will also discuss gaps in our knowledge about IL17RD function to provide insight into opportunities for future investigation.

**Video Abstract**

**Video Abstract**

## Background

Interleukin-17 receptor D (IL17RD), originally designated as Sef (similar expression to fgf), was discovered during a high throughput in situ hybridization screening of zebrafish and frog embryos [1, 2]. *Sef* expression was demonstrated to be regulated by fibroblast growth factor (FGF) signaling during zebrafish, frog and chick development, wherein it was shown to regulate FGF induced Extracellular Regulated Kinase (ERK) activation and associated developmental phenotypes, thus implicating SEF as a feedback inhibitor of FGF signaling [1–3]. Subsequently mouse and human orthologs of S*EF (IL17RD)* were also determined to regulate FGF as well as several other signaling pathways to regulate a wide range of biological process ain of interleukin 17 receptors (SEFIR, SEF/IL17R domain), *SEF* was designated as *IL17RD* [1, 2, 4]. In this review, we will elaborate upon the current field of knowledge with respect to SEF (IL17RD), and provide a prospective into areas of future research. As a note to the reader, we will use the terms SEF and IL17RD interchangeably based upon their usage in the original citation, for the remainder of this review.

### Identification and characterization of *SEF (IL17RD)* homologs

Homologs of *IL17RD* have been described in many vertebrates, chordates, and an *IL17R* ancestral gene with a SEFIR/TIR domain was identified in *C. elegans* [6, 7]. *Sef* (*Il17rd*) was initially identified and characterized as playing a role in development of *D. rerio* and *X. laevis* embryos, in part by regulating FGF signaling. *Sef* exhibited overlapping patterns of spatiotemporal expression with *fgf3*, *fgf8*, *fgf17* and *spry4* in these embryos, suggesting that these genes function together in a common pathway, constituting the *fgf* synexpression group [1, 2, 8]. Injection of *fgf8* RNA into zebrafish embryos resulted in *sef* expression throughout the embryo, whereas its normal expression is highly restricted to zones of endogenous Fgf signaling, including the midbrain-hindbrain boundary and limb buds; and inhibition of Fgf signaling using antagonists and dominant negative constructs suppressed *sef* expression (1, 2). Injection of *sef* antisense morpholino oligonucleotides recapitulated phenotypes consistent with ectopic expression of *fgf8*, whereas overexpression of *sef* in frog and zebrafish produced a phenotype similar to the expression of a dominant negative Fgfr [1, 2]. This was accompanied by concomitant dysregulation in expression of *fgf* responsive genes, whereas the expression of target genes of other pathways remained unaffected, suggesting that Sef functions as an antagonist of Fgf signaling [2]. Sef was also shown to regulate Fgf-induced responsiveness of blastemal cells to regulate position dependent growth in a model of adult zebrafish fin amputation, implicating a role for Sef in tissue regeneration [9]. In the chick *(G. gallus)*, *SEF* is expressed in limb bud mesoderm beneath the apical ectodermal ridge (AER) in a region called “progress zone” during development, and removal of the AER eliminates *SEF* expression. *SEF* expression was restored in limb bud mesoderm by implanting FGF2 coated beads whereas ectopic *SEF* expression was induced by implantation of FGF2 and FGF4 coated beads [3]. In this context, SEF was shown to regulate limb morphogenesis and digit specification [3, 10]. Whereas in mice *(M. musculus)*, expression of *Sef* showed extensive overlap in expression with *Fgf8, Fgf15, Fgfr1* and *Spry2* during mouse brain development [11, 12]. In this context, SEF was shown to function synergistically with SPRY2 to regulate midbrain-hindbrain patterning in the developing mouse embryo [13]. Although loss of *Sef* did not result in severe phenotypes developmentally, adult *Sef−/−* mice exhibited defects in the responsiveness of the brainstem to auditory stimuli. In this context, SEF was expressed in the astrocytes of the cochlear nucleus, wherein it functioned to regulate FGF signaling from the adjacent rhombic lip [12]. In humans *(H. sapiens)*, the expression pattern of *SEF* was determined to be more extensive to several organs and tissues, wherein it was found to regulate FGF2 induced activation of multiple downstream effector pathways and cellular processes, as well as regulate signaling downstream of other RTK and inflammatory cytokines (see below) [4, 14, 15]. For information on sequence homology and evolutionary conservation of SEF amongst species, the reader is directed elsewhere [16].

### Structure and conformation of IL17RD (SEF)

**Fig. 1:**
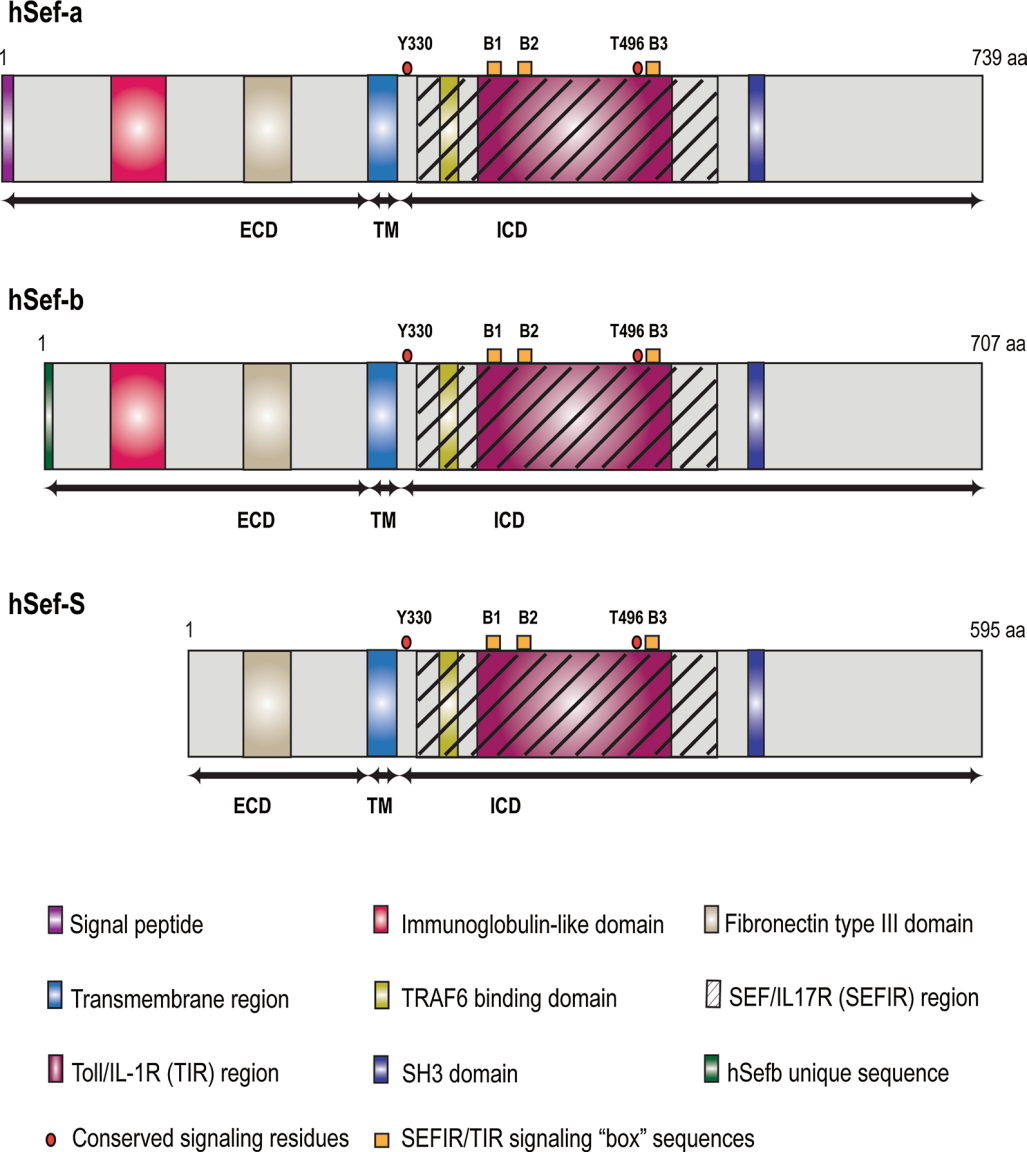
Visual representation of putative structural and functional domains encoded by Sef (IL17RD) isoforms. The structure of Sef is comprised of an extracellular domain, a transmembrane domain and an intracellular domain which are delimited by the arrows. hSEF-a (IL17RD) contains a signal peptide sequence (residues 1–26) immediately upstream of the extracellular domain (residues 27–299). Within the extracellular domain are conserved domains, including an immunoglobulin-like domain (residues 89–126) and a fibronectin type III domain (residues 199–281). The extracellular domain is followed by a short transmembrane domain (residues 300–319). The intracellular domain (residues 320–739) contains a Sef/IL17R homology region (SEFIR) (residues 335–564) which contains a TRAF6 binding subdomain (residues 348–352) and a Toll/IL1R (TIR) subdomain (residues 355–508). The SEFIR domain is followed by a putative SH3 domain (residues 567–578) followed by a short cytoplasmic tail (residues 579–739). The conserved tyrosine 330 (Y330) and threonine 496 (T496) residues are indicated, and the three short sequence “boxes”, indicated as B1 for Box1 (residues 357–362), B2 for Box2 (residues 377–381) and B3 for Box3 (residues 500–503). hSEF-b is a cytosolic variant that lacks first 42 residues including the signal peptide, which are replaced by 10 new amino acids. hSEF-S is translated from an alternate initiation codon downstream of the hSEF-a initiation codon, and therefore lacks the signal peptide and immunoglobulin domain thus remaining in the cytoplasm

Structurally, human SEF is a type I transmembrane protein that is evolutionarily conserved among vertebrates and is encoded by a single locus on chromosome 3p14.3 in humans [1, 2, 4]. The mRNA consists of 13 exons encoding a polypeptide product of 739 amino acids comprised of a 26-residue amino terminal signal peptide, followed by a 272 amino acid extracellular domain, a short transmembrane domain consisting of 20 amino acids, and a 420 amino acid intracellular domain [4] (Fig. 1). The extracellular domain of SEF consisting of a signal peptide, an immunoglobulin-like domain and a fibronectin type III repeat. This domain of SEF contains seven potential sites for N-linked glycosylation, and eight conserved cysteine residues which are hypothesized to play an important role in maintaining its secondary structure. The specific location of these sites is described elsewhere [4]. The extracellular region is followed by a short, single span transmembrane domain, and a cytoplasmic domain containing a highly conserved tyrosine residue (Tyr330) in the juxtamembrane domain. This invariant residue belongs to the YXXø consensus motif (where X is any amino, and ø is a hydrophobic amino acid) and is required for its inhibitory activity to FGF signaling in *Xenopus* embryogenesis [1, 2, 4, 17] and inhibition of Nuclear Factor kappa light chain enhancer of activated B cells (NF-κB) activity in mice and humans [18]. Additionally, this residue was also shown to function as a sorting motif to regulate the subcellular localization of SEF in Human embryonic kidney (HEK293) cells, and consequently of the FGFR-SEF complex [17]. This is followed by a highly conserved SEFIR segment, designated based on sequential and structural homology with the cytoplasmic domain of interleukin-17 receptors. The SEFIR domain contains a putative TNF receptor associated factor 6 (TRAF6) binding site; and a TIR (Toll/Interleukin-1 Receptor) subdomain containing three conserved putative TIR binding motifs or “boxes”, and a conserved threonine residue (Thr496) which is essential for IL17RD regulation of Toll-like receptor 4 (TLR4) signaling. Since the SEFIR and TIR domains share a high degree of structural homology, they were classified as members of the STIR superfamily. The SEFIR domain is followed by a proline rich putative Src homology 3 (SH3) binding domain [1, 2, 4, 19–21]. A visual representation of Sef (IL17RD) protein containing the putative interactive domains is shown in Fig. 1.

Within the cell, SEF (IL17RD) expression has been localized mainly to the plasma membrane [4, 5] although its expression has also been observed in the Golgi apparatus [22, 23] and in early and recycling endosomes (perinuclear structures) [24] in overexpression systems. Physiologically, SEF has been shown to exist in monomeric, dimeric as well as oligomeric forms, showing a preference for oligomerization [4, 18]. In its dimeric form, SEF exists as a homodimer as well as in heterodimeric forms with FGFR1 (through its extracellular, transmembrane and intracellular domain), FGFR2 (via its extracellular, transmembrane and intracellular) [25], IL17RA (predominantly through its SEFIR domain) [26], TNFR2 (via its extracellular domain) [27] TLR3 (partly through its SEFIR domain) and TLR4 (partly through its SEFIR domain) and with Epidermal Growth Factor Receptor (EGFR) [24]. The homomeric interactions between SEF molecules were independent of the SEFIR domain of IL17RD, indicating that this domain might play a role in facilitating heterotypic interactions with other SEFIR containing receptors, whereas the transmembrane domain might facilitate homotypic interactions [18].

### Isoforms of *SEF (IL17RD)*

The human *SEF (IL17RD)* gene give rise to several splice variants and transcripts that use alternate translation initiation sites to generate transmembrane as well as cytoplasmic isoforms of *SEF (IL17RD)*. One transmembrane and two cytoplasmic *SEF* isoforms have been identified in humans and are designated as *hSEF-a, hSEF-b* and *hSEF-S* respectively [19, 28, 29] (Fig. 1). The *hSEF-a* isoform encodes a polypeptide product of 739 amino acids that is heavily glycosylated and is localized to the plasma membrane by virtue of its signal peptide sequence [4]. The *hSEF-b* isoform lacks the secretory signal peptide present in the full-length form of *SEF*, but instead has a unique 10N-terminal amino acid sequence resulting in a product of 707 amino acids. This isoform does not undergo any significant post-translational modifications, despite having similar putative sites for glycosylation as hSEF-a [28]. *hSEF-b* is expressed at significantly lower levels than *hSEF-a* due to factors such as translation initiation from a CUG initiation codon located within a weak Kozak sequence and lack of a poly(A) tail, which may affect mRNA stability [28, 29]. Similar to *hSEF-b*, the *hSEF-S* isoform also lacks the signal peptide; however, it is translated from an alternative initiation site located within an optimal Kozak sequence to encode a shorter product of 595 amino acids. Thus, *hSEF-a, hSEF-b* and *hSEF-S* originate from alternative translation initiation start sites within the *hSEF* transcript [19, 29]. A visual representation of the proteins encoded by the different *SEF* isoforms is shown in Fig. 1.

### Expression pattern of IL17RD (SEF)

Several studies have performed expression analyses to characterize SEF levels in various organs. The *hSEF-a* isoform is more ubiquitously expressed in various organs. Predominantly, *hSEF-a* has been shown to be expressed in epithelial tissues such as gonads, breast, small intestine, eyes and skin keratinocytes. High levels have also been observed throughout the brain (including hypothalamus and pituitary), endothelial cells, kidney, bone, spinal cord, skeletal muscle, heart and nerves (Schwann cells) [4, 28, 30, 31]. Moderate expression of *SEF* has been observed in small intestine, tonsils, spleen, adenoids and the liver whereas low levels have been observed in the adrenal gland, peripheral blood leukocytes, smooth muscle cells, lung, bladder, pancreas, adipose and spleen [4, 28]. With respect to *hSEF-b*, the expression pattern is more restricted, with high levels observed in thyroid and testes, moderate in the brain and low levels in endothelial cells [28]. The expression of the *hSEF-S* has not been extensively characterized, with the adrenal medulla, which expresses low levels of *hSEF-a* and no *hSEF-b*, being the only known organ system [19, 28]. The expression of the different isoforms is thought to be regulated by cell type specific transcriptional machinery governed by epigenetic mechanisms, such as the Polycomb (PcG) group of proteins [16, 32].

### SEF (IL17RD) regulation of cellular signaling pathways

In the past few years, the role of IL17RD has been expanded to include several different pathways within its interactive network. Summarily, IL17RD can be considered as a signaling node acting as a scaffold for the assembly of various receptor complexes and their respective interacting proteins to generate a variety of signaling outputs.

### SEF (IL17RD) regulation of RTK signaling pathways

#### FGF

**Fig. 2:**
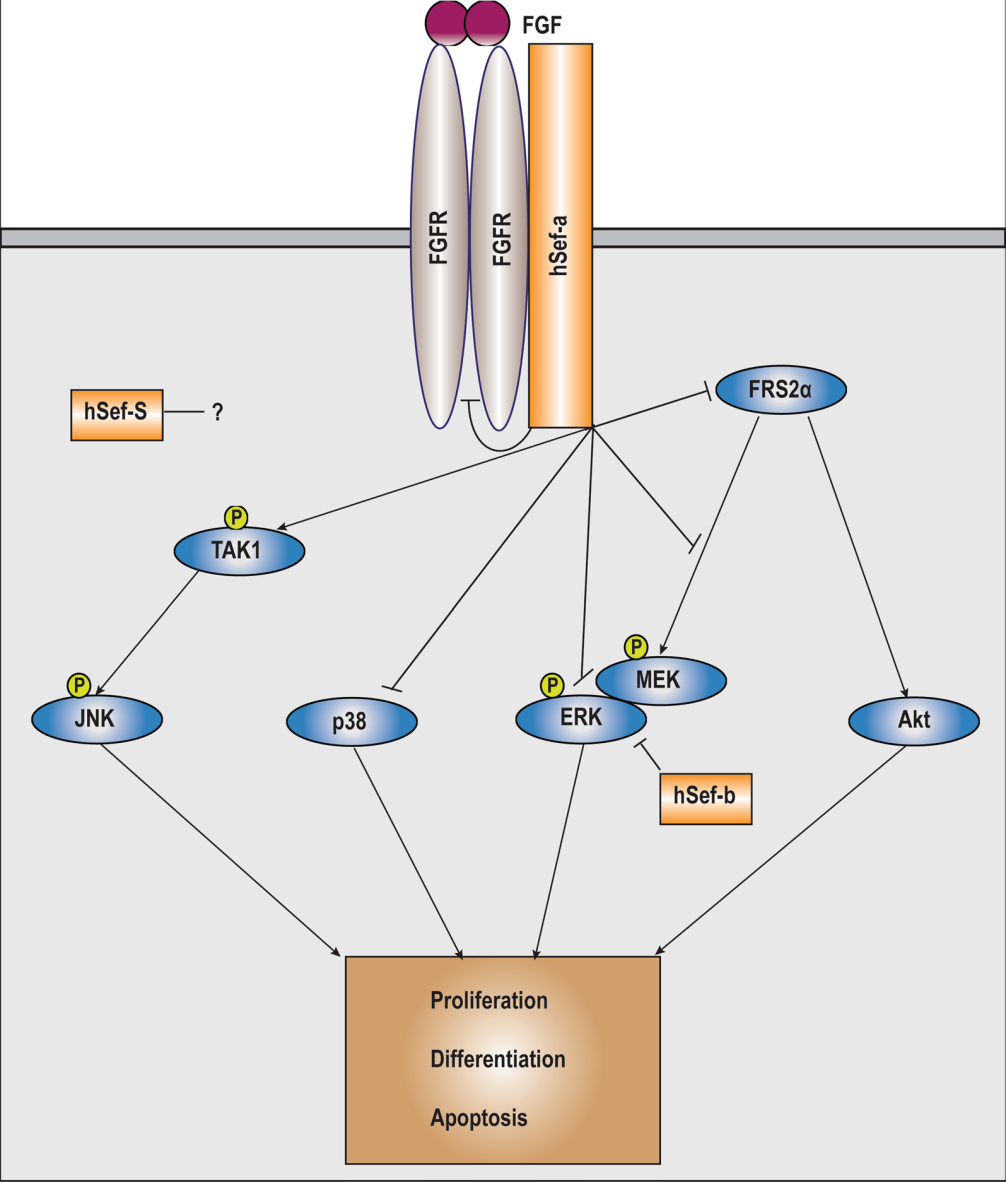
Schematic diagram depicting modulation of the FGF signaling cascade by different isoforms of hSEF. The transmembrane form of Sef (hSEF-a), physically associates with FGFR1 and FGFR2 to restrict FGFR kinase activation and subsequent FRS2α phosphorylation upon FGF stimulation. By inhibiting FGFR mediated FRS2α tyrosine phosphorylation as well as subsequent adaptor protein activation, the ERK and AKT pathways are attenuated. hSEF-a is also reported to bind to the MEK-ERK complex without inhibiting activation of its components, but impedes dissociation and nuclear translocation of ERK and thus activation of its downstream targets. Conversely, the cytosolic hSef-b isoform specifically restricts ERK phosphorylation. hSef-a also regulates the activation of the p38 MAPK pathway in a cell type specific manner; and promotes the activation of the JNK pathway by associating with TAK1. The cytosolic hSef-S isoform regulates cellular proliferation through an undefined mechanism

All three isoforms of SEF have been shown to bind to FGFR by coimmunoprecipitation analysis and affect the activation of different effector molecules downstream of FGF [5, 28, 29]. The transmembrane form of SEF (hSEF-a) has been determined to inhibit the FGF signaling pathway to suppress its mitogenic response, however its point of action in the FGF signaling pathway has been debated, with it having been shown to interact either at the level of FGFR and fibroblast growth factor receptor substrate 2α (FRS2α) [2, 5], at the level of RAS [33], or downstream of RAF1 at the level of dissociation of the Mitogen Activated Protein Kinase Kinase (MEK)-ERK complex into the nucleus [22] and at the level of ERK phosphorylation [1, 4] (Fig. 2). Further, one of the cytosolic isoforms of SEF, hSEF-b, was shown to restrict the proliferative response of FGF specifically at the level of ERK activation, by interfering with its phosphorylation [14, 28]. Studies from our laboratory revealed that the transmembrane form of mouse Sef (mSef) co-immunoprecipitated with FGFR1 to inhibited FGF induced RAF, MEK and ERK activation, as well as of AKT [5]. mSEF was shown to inhibit ERK activation induced by a constitutively active FGFR1, but not constitutively active RAS, suggesting that this isoform acts upstream of RAS and likely by directly binding to FGFR1 and FGFR2. The interaction of SEF with FGFR is independent of factors such as ligand stimulation, FGFR dimerization and kinase activity [5]. Further receptor crosslinking analysis determined that the extracellular as well as the transmembrane domains of SEF interact with the transmembrane FGF receptor, with most of the interaction facilitated by the transmembrane region of SEF [5]. Thus, the interaction between FGFR1 and SEF might occur at multiple sites on both receptors. It is currently unknown whether mSEF interacts with or inhibits signaling by FGFR3 or FGFR4. However, expression of *Sef* was observed at several sites in the developing mouse embryo coinciding with the expression of known ligands of FGFR3c and FGFR4 (FGF17 AND FGF15/FGF9), suggesting that IL17RD might interact with these receptors [11, 14]. It is also unknown whether IL17RD affects the recruitment of other FGFR adapter proteins such as CRK and SHC which might explain the previously observed discrepancies observed in recruitment and phosphorylation of adapter proteins and consequent pathway activation.

In response to FGF stimulation, over expression of mSEF resulted in decreased proliferation and cellular viability in murine NIH3T3 cells and this was associated with a decrease in tyrosine phosphorylation of FGFR1; as well as of the FGFR signaling adaptor protein FRS2α [5]. The mechanism of interaction between FRS2α and SEF remains to be defined, and may occur via interaction with FGFR1 by masking of the valine-threonine dipeptide region in the juxtamembrane region of FGFR1 required for binding to FRS2α. Alternatively, SEF could act as a brake between FGFR1 and FRS2α to regulate tyrosine phosphorylation. This is supported by the observation that hSEF-a is itself phosphorylated at several tyrosine residues, including Y330, in a manner dependent on FGFR kinase activity [14]. Thus, SEF inhibits the FGF signaling cascade by inhibiting the phosphorylation of the FGF receptor and itself undergoing phosphorylation in this process either through FGFR or by recruitment of an intermediate kinase [14, 25]. However, the specific role of the intracellular conserved tyrosine 330 residue located in the intracellular domain in this context remains to be clarified, due to conflicting reports of its effect on the activation of the ERK pathway. In zebrafish, mutation of tyrosine 329 to phenylalanine (corresponding to tyrosine 330 in mice and humans) showed phenotypes consistent with enhanced Fgf signaling [2], suggesting that this residue was indispensable for Sef inhibitory action on FGF signaling, whereas in humans this mutation was shown to have no effect [22] or to conversely enhance the suppressive effect of SEF on FGF signaling [17]. It was also observed that deletion of residues 356–395 of the SEFIR domain, located proximal to the Y330 domain abolished the inhibitory effect of SEF on FGF signaling in HEK293 cells, however the role of this domain has also been debated [18, 22]. These discordances can be partly attributed to factors such as differences in relative expression levels and possible variations in folding patterns between mutant and wild type SEF proteins, however, further investigation is required. Taken together, these results suggested that suppression of mitogenesis by SEF is mediated predominantly through its intracellular domain [18]. The intracellular domain of SEF was also shown to inhibit the differentiation of rat pheochromocytoma (PC12) cells into neurons by suppression of sustained ERK signaling [19]. However, studies from our laboratory have shown that the intracellular domain of SEF contributes only partially to mSEF inhibitory activity, with the extracellular and transmembrane domain of IL17RD functioning as the predominant suppressors of FGF signaling activity in HEK293 and NIH3T3 cells [5, 25]. A possible explanation for this difference in results is that the extracellular and transmembrane domains of SEF may modulate immediate ERK activation, with the intracellular domain perpetuating the prolonged inhibitory effect to FGF stimulation. Collectively, these results indicate that all three regions of SEF (IL17RD) contribute to its inhibitory effect on FGF signaling, in a cell type specific manner.

Although hSEF-a suppressed FGF2 induced mitogenesis in HEK293 and NIH3T3 cells by downregulating the expression of CYCLIND1, the downstream mechanism of this inhibition differs by cellular context. In HEK293 and HeLa cells, ectopic expression of hSEF-a inhibited cell proliferation by restricting the activation of ERK, whereas in NIH3T3 cells this was shown to occur through an ERK-independent and AKT dependent mechanism [14]. Concurrently, SEF also promotes apoptosis of NIH3T3 cells in response to FGF2 in a manner dependent on activation of p38 Mitogen activated protein kinase (p38 MAPK) in these cells, whereas it suppresses the phosphorylation of p38 MAPK in apoptotic endothelial cells [14, 25]. Although mSEF did not affect the activation of the JNK pathway in endothelial cells, it was shown to regulate HEK293T cell survival in a JNK dependent manner by binding to Transforming growth factor beta Activated Kinase 1 (TAK1) via its cytoplasmic tail leading to phosphorylation of c-JUN via intermediate activation of Mitogen Activated Protein Kinase Kinase (MKK4) [25, 34]. Furthermore, the hSEF-S isoform was shown to regulate FGF induced mitogenesis in an ERK independent manner [29]. These results indicate that the varied mode of action of SEF on downstream signaling cascades might be attributable to cell type specific mechanisms governed by cellular source of origin (epithelial versus mesenchymal), contextual factors as well as isoform specific effects [16, 28, 34].

Recent data indicate that SEF might also regulate FGFR trafficking and turnover to regulate FGFR signaling at multiple levels. Overexpression of a mutant intracellular form of mSEF was associated with a decrease in total FGFR levels in HEK293 cells, indicating that this domain may regulate the intracellular trafficking and possible degradation of the FGF receptor [25]. The conserved tyrosine residue (Y330) which is present in a key subcellular localization motif in the intracellular domain, was shown to promote endocytosis of FGFR-SEF complex [17], indicating that FGF-induced phosphorylation of this residue [14] might render the FGFR-SEF complex more susceptible to receptor mediated endocytosis and subsequent ubiquitination [24]. Consistent with this, SEF and FGFR have been shown to exhibit a reciprocal pattern of expression in pathological conditions [35, 36], thus rendering an additional level of complexity to the SEF-FGFR relationship.

#### PDGF

Although expression of mSEF was shown to be ineffective towards activation of ERK induced by Platelet Derived Growth Factor (PDGF) [5], one of the cytosolic isoforms of SEF, hSEF-b was shown to inhibit PDGF induced ERK activation and consequent proliferative response in NIH3T3 cells [28]. Replacement of the transmembrane domain of SEF with a PDGF receptor (PDGFR) transmembrane domain did not affect its response to PDGF [25], suggesting that hSEF-b action on PDGF induced ERK activation occurs due to its effect on common intracellular pathways involved in the process as opposed to a specific interaction with the PDGF receptors, although further experiments are needed to verify this. Given the restrictive expression of hSEF-b in mammalian tissues, the functional consequences of hSEF-b inhibition of PDGF/ERK pathway still remain to be determined [28].

#### NGF

SEF has also been shown to inhibit differentiation of PC-12 cells in response to Nerve Growth Factor (NGF) at both physiological as well as high doses of NGF, probably through restriction of sustained ERK activation [19]. However, it remains unknown whether SEF exerts this effect by interacting with the receptor tyrosine kinase tropomyosin receptor kinase A (TrkA) and modulating the recruitment of FRS2α, or with the TNF superfamily receptor LNGFR (p75NTR), or with both. Alternatively, SEF might interrupt heterodimerization of the TrkA/LNGFR high affinity receptor complex to regulate downstream signaling [19].

#### EGF

SEF regulation of EGF signaling has been ambiguous, since it has been shown to either weakly inhibit or enhance EGF signaling, albeit by different mechanisms. Torii et al., showed that SEF suppressed EGF signaling downstream of MEK by constraining the nuclear translocation of activated ERK without affecting its phosphorylation [22], whereas Ziv et al., showed that SEF inhibits EGF signaling by inhibiting the phosphorylation of ERK [14]. On the other hand, Ren et al., showed that SEF binds to EGFR in an EGF dependent manner and promote endocytic receptor recycling, consequently enhancing ERK activation [24]. A possible explanation for the differences in the results might be that SEF functions as a weak antagonist of EGF signaling, inhibiting ERK activation at lower doses whilst at higher doses it is either ineffective or undergoes ubiquitination by c-CBL in a manner similar to that of Sprouty [37–39], however this remains to be determined experimentally.

### SEF (IL17RD) regulation of inflammatory signaling pathways

#### IL17A

**Fig. 3:**
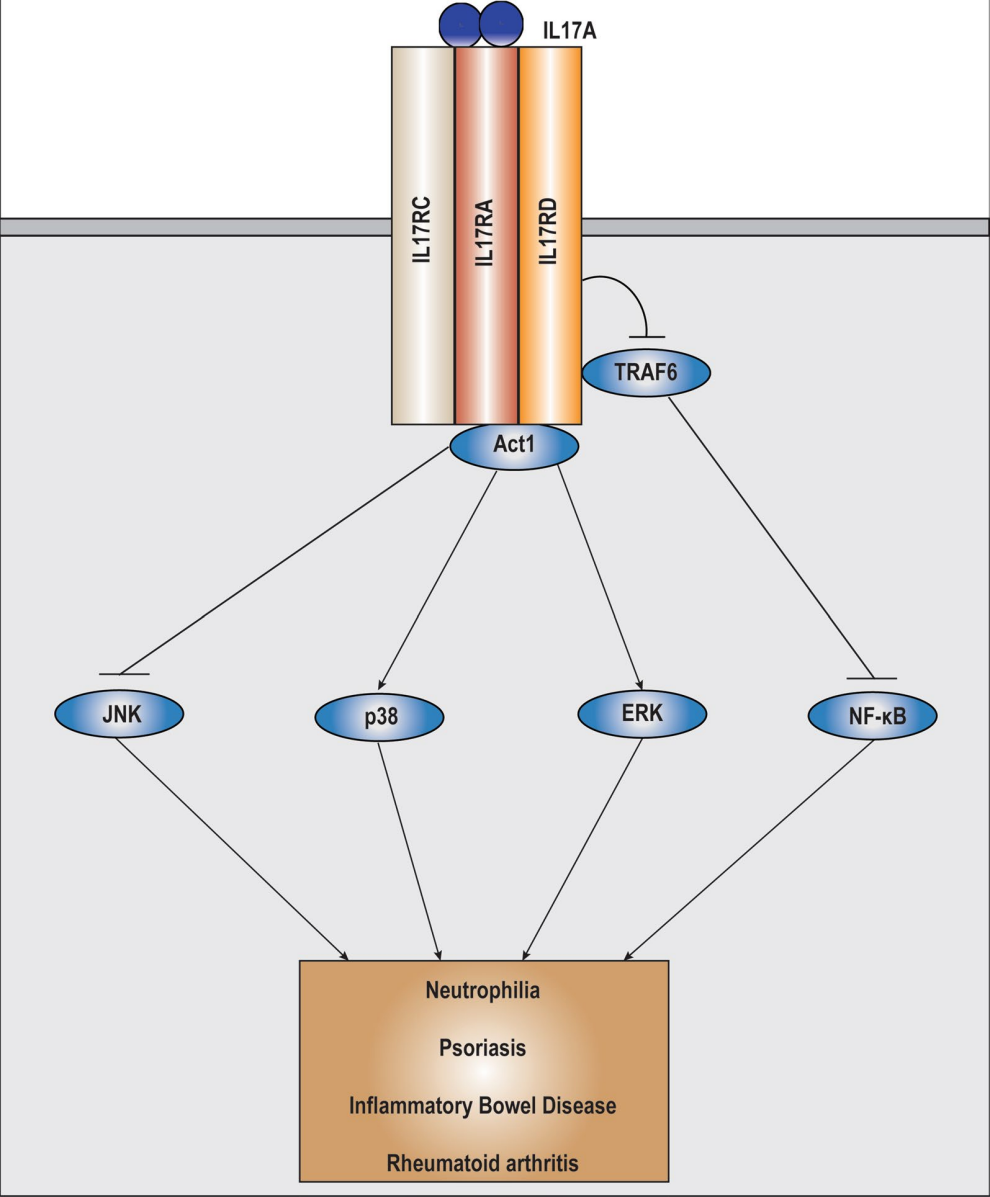
Schematic diagram depicting IL17RD (Sef) regulation of the IL17 signaling cascade and associated disease relevance. IL17RD binds to IL17RA in a cell type dependent manner to regulate IL-17 signaling. Upon binding of IL17A to the IL17RA/IL17RC/IL17RD receptor complex, IL17RD modulates the recruitment of adapter proteins ACT1 and TRAF6 to the receptor complex. IL17RD subsequently regulates the polyubiquitination of TRAF6; as well as the activation of predominantly the p38 MAPK, and the ERK, JNK and NF-κB pathways to differentially regulate expression of target genes. Consequently, genetic perturbations and disruptions in IL17RD expression have been associated with several inflammatory diseases and immunomodulatory phenotypes associated with the IL17 signaling superfamily

Although SEF was identified and named due to its association with the FGF signaling, it was designated as a member of the IL17 receptor family (IL17RD) based on sequence homology with other IL17 receptors [2, 4] Despite this, the role of IL17RD (SEF) with respect to IL17 signaling initially remained unexplored. However, Rong et al., showed that IL17RD mediated IL17A induced downstream pathway activation [15]. IL17RD was capable of modulating IL17 signaling in its homodimeric as well as in a heterodimeric state with IL17RA [15] (Fig. 3). The interaction between IL17RA and IL17RD was shown to occur independent of IL17 stimulation through multiple sites on both receptors, with most of the interaction occurring via the SEFIR domain [15, 26]. Additional studies showed that IL17RD functions as modulator of IL17 induced downstream effector pathways in primary mouse embryonic fibroblasts (MEFs) and bone marrow derived macrophages (BMDMs) [26], and as a physiological receptor for IL17A to differentially regulate expression of IL17A target genes in keratinocytes [40]. Furthermore, expression of IL17RD has been shown to be associated with T Helper 17 (Th17) cell differentiation and polarization under physiological as well as pathological conditions. Since the inflammatory cytokine IL6, a key downstream target of the IL17A-IL17RD pathway contributes to sustained Th17 differentiation, it can be postulated that IL17RD acts in a feed forward loop in the context of IL17A signaling; however further studies are required in this regard [26,41,42].

With respect to the IL17A–IL17RA/IL17RC signaling pathway, IL17RD was shown to weakly associate with the key adapter proteins ACT1 (through its SEFIR domain) and TRAF6 (probably via its TRAF6 binding domain), suggesting that it might function to modulate the spatial interaction between ACT1, TRAF6 and IL17RA in the context of canonical pathway activation via its intracellular domain (Fig. 3). Concurrently, loss of IL17RD decreased IL17 induced activation of the p38 MAPK pathway to consequently downregulate the expression of Macrophage Inflammatory Protein 2 (MIP-2). This was accompanied by a slight increase in the activation of the ERK pathway and no significant effect on the activation of the JNK pathway [26]. However, in the same context, loss of IL17RD significantly enhanced ACT1 mediated ubiquitination of TRAF6 to increase IL17 induced NF-κB activation and upregulate the expression of IL6. Further, IL17RD did not significantly affect the TRAF2/TRAF5 component of the pathway, implying that IL17RD differentially regulates the IL17A-IL17RA/IL17RC signaling axis [26]. In another cellular context, loss of IL17RD in keratinocytes significantly decreased IL17A induced activation of p38 MAPK and JNK to differentially regulate IL17A induced chemokine expression. This was accompanied by a slight decrease in ERK activation and a slight increase in NF-κB activation [40]. In this model, IL17RD showed preferential binding affinity for IL17A in its heterodimeric form with IL17RA but had significantly lesser affinity for IL17RA compared to IL17RC, suggesting that IL17RD might function as an accessory receptor of the IL17RA/IL17RC complex in this context. Alternatively, IL17RD might function as a scaffold to mediate the interaction between IL17RA and IL17RC interaction [40]. Although the interaction of IL17RD and IL17RC still remains to be investigated, IL17RD was shown to regulate IL6 production in response to IL17F stimulation in primary MEFs, suggesting that IL17RD might interact with IL17RC [26]. It is also possible that IL17RD might be capable of binding to other receptors in the IL17 superfamily, since the interaction between IL17RA and IL17RD was shown to be mediated predominantly via the SEFIR domain, which is conserved amongst all IL17Rs [26, 40]. Indeed it was observed that IL17RD associated with IL17RB in an overexpression model in HEK293 cells [15]. Furthermore, it was also shown that *Il17rc−/−* and *Il17rd-/-* mice only partially recapitulated the psoriatic phenotype of *Il17a/f* double knockout mice, suggesting that IL17RD might also regulate signaling downstream of the IL17A/F heterodimer in addition to IL17A [40]. Taken together, these results suggest that IL17RD associates with the IL17RA/IL17RC receptor complex to bind to and modulate IL17A signaling in a cellular context dependent manner, and may also associate with other IL17 receptors to affect signaling pathways downstream of other IL17 cytokines.

### TIR signaling family

**Fig. 4:**
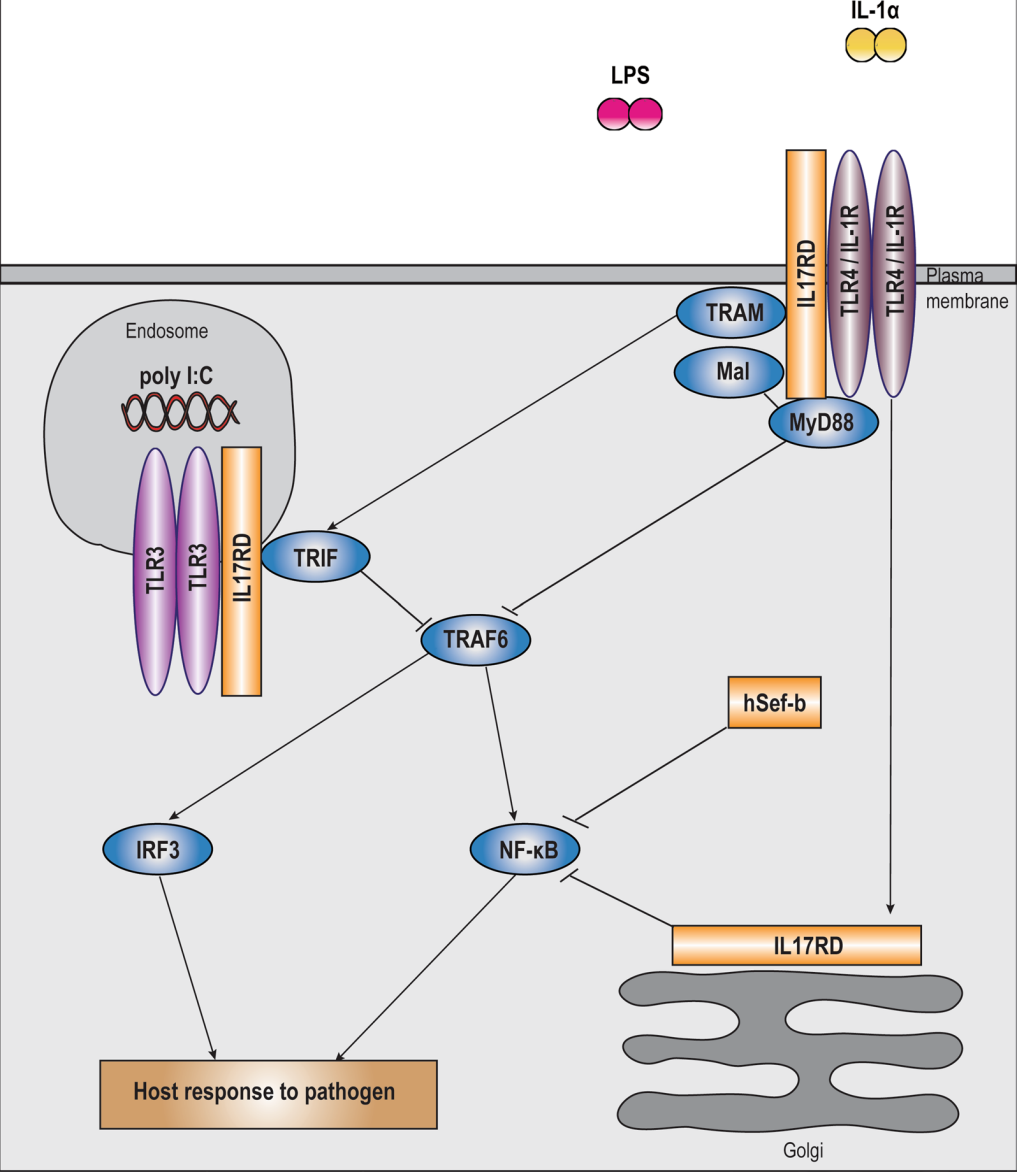
Schematic diagram depicting IL17RD (Sef) modulation of the TIR signaling pathway. IL17RD regulates TIR signaling by interfering with recruitment of upstream and downstream adapter proteins to modulate host immune response. In TLR signaling, IL17RD binds to plasma membrane TLR4 and endosomal TLR3 in resting cells. Upon recognition of extracellular and intracellular microbial components such as LPS and poly I:C, IL17RD associates with adapter proteins MyD88, Mal, TRIF and TRAM to modulate their interaction with TRAF6. Therefore, IL17RD indirectly suppresses the ubiquitination of TRAF6 to inhibit the activation of the canonical NF-κB and non-canonical IRF3 pathways and expression of target genes. With respect to signaling downstream of IL1 ligands from the TIR family, IL17RD regulates activation of the NF-κB pathway downstream of IL1α binding to IL1R, by associating with the IKKβ, IKBα and p50 subunits at the Golgi and restricting pathway activation

The SEFIR domain of IL17RD contains a region which shares a high degree of sequential and structural homology with the intracellular domains of receptors from TIR families, due to which IL17Rs and TIRs were reclassified as members of the STIR superfamily [20] (Fig. 1). In particular, the SEFIR region of IL17RD shows sequence homology with TIR in highly conserved short sequence motif “boxes” 1 and 2 (out of 3), all of which are known to be functionally important (see Fig. 1). Boxes 1 and 2 have been shown to be critical for obligatory homotypic interactions between TIR family receptors, whereas box 3 was hypothesized to play a key role in determining subcellular localization, thereby creating an interactive TIR subdomain within the SEFIR domain [20]. IL17RD has been shown to function as an integral component of the TIR signaling cascade by functioning at the level of interaction with adapter proteins as well as at the level of activation of downstream pathway effector molecules (Fig. 4).

#### TLR

In the context of TLR signaling, IL17RD was shown to associate with the intracellular TLR3 as well as the membranous TLR4 receptors partly via the SEFIR domain to negatively regulate downstream pathway activation. Upon ligand recognition, IL17RD binds to key TLR downstream adapter proteins MyD88 adaptor-like (Mal), Myeloid Differentiation primary response protein 88 (MyD88), TIR-domain-containing adapter-inducing interferon β (TRIF) and TRIF-related adaptor molecule (TRAM) through its conserved SEFIR domain in a time dependent manner in mice and humans [21]. Specifically, IL17RD and MyD88 bind to each other via their respective Box3 sequences to inhibit TRAF6-MyD88 interaction and TRAF6 polyubiquitination, to consequently suppress activation of the canonical NF-κB and the non-canonical IRF3 and IRF7 pathways, and associated target gene expression (Fig. 4). Thus, IL17RD regulates TLR pathway activation in an indirect manner independent of its previously known direct binding capacity to TRAF6 and TAK1 [15, 21, 26, 34]. The functional capacity of the IL17RD-Myd88 complex to modulate TLR signaling is specifically localized to a threonine residue located proximal to the Box3 region (Thr496). However, the kinetics of prospective Thr496 post translational modifications upon TLR ligand stimulation still remains to be deciphered to provide insight into interaction of the IL17RD/MyD88 complex with intermediate adapter proteins Interleukin 1 Receptor Associated Kinase 1 (IRAK1) and IRAK4, and consequently with TRAF6. Furthermore, the functional significance of the interaction of MyD88-IL17RD-TRAF6 interaction in this context still remains to be delineated. Given that the TRAF6 domain lies juxtaposed upstream of the TIR subdomain within the IL17RD SEFIR domain (Fig. 1), it is possible that the interaction between IL17RD and MyD88 might mask the TRAF6 binding site within the IL17RD SEFIR domain [21]. Additionally, the specific regions within SEFIR mediating its interaction with adapter proteins Mal, TRIF and TRAM also needs to be elucidated [21]. Further, since the SEFIR domain was shown to play only an accessory role in heterodimerization with TLR3 and TLR4 probably due to absence of essential intra-TIR binding motifs, the additional regions within SEF contributing to its interaction with TLRs remain to be identified [16]. To synopsize, in the context of TLR signaling, the conserved boxes 1 and 2 within the IL17RD SEFIR domain partially facilitate homotypic interactions with TLR receptors, whereas the divergent Box 3 interacts with MyD88 to regulate activation of downstream pathways.

#### IL1α

With respect to IL1α signaling, studies have focused on the role of IL17RD specifically at the level of NF-κB activation. Fuchs et al. performed in silico analysis to implicate IL1α and Tumor Necrosis Factor α (TNFα) as prospective cytokine regulators of IL17RD expression and identified the presence of putative NF-κB response elements on the IL17RD promoter [23]. Using in vitro expression analysis in NIH3T3 cells, they demonstrated that activation of c-Jun N-terminal kinase (JNK) and NF-κB pathways in response to IL1α stimulation induced the expression of IL17RD in NIH3T3 cells, which subsequently functions as a negative regulator of the activation of the canonical NF-κB pathway (Fig. 4). Molecularly, IL17RD binds to and masks the C-terminal Nuclear localization sequence (NLS) of the p50 subunit of the NF-κB heterodimer via its conserved tyrosine residue to constrain the nuclear translocation of the NF-κB complex, thereby establishing IL17RD as a novel component of the NF-κB:IκB complex [23]. Further analysis using in vitro cell binding assays established that multiple additional regions in the intracellular domain of IL17RD mediate this interaction, including the SEFIR domain and the carboxy terminal tail region, through formation of a putative p50 binding subdomain. IL17RD was also demonstrated to bind with other components of the NF-κB pathway, including Inhibitory Subunit of NF Kappa B Alpha (IκBα) and IκB kinase (IKKβ), partially through the SEFIR domain and via residues 321–395 respectively [18]. Accordingly, the hSEF-b isoform has been shown to repress activation of the NF-κB pathway, probably by binding to the NF-κB subunits through the intracellular domain which is common to both isoforms [43]. Thus, SEF (IL17RD) was defined to have a unique role as a scaffolding protein for components of the canonical NF-κB pathway [18]. This function of IL17RD was shown to occur primarily in the Golgi apparatus in a manner independent of post translational modifications [23]. Since IL17RD does not affect the phosphorylation on IKKα/β, it remains to be determined whether IL17RD itself undergoes phosphorylation at Ser/Thr residues by IKKα/β to restrict the phosphorylation and probable ubiquitination of p50 and IκBα.

#### TNFα

Similar to its response to IL1α, IL17RD was shown to inhibit TNFα induced translocation of the activated NF-κB complex in NIH3T3 cells [23]. In contrast, Yang et al. determined that IL17RD potentiated TNFα induced NF-κB activation in a TRAF2 dependent manner in human and mouse kidney cells by weakly associating with the extracellular domain of TNFR2 through its own extracellular domain. However, this phenomenon was restricted to tissues expressing TNFR2, whereas cells lacking TNFR2 expression showed a suppression of TNFα induced NF-κB activity, similar to results obtained by Fuchs et al. [27]. This suggests that binding of TNFR2 to IL17RD via the extracellular domain induces a conformational change in the oligomerically bound IL17RD within the Golgi to mask the exposed TIR adapter protein/NF-κB binding sites, resulting in release of the bound subunits to induce NF-κB hyperactivation.

##### WNT

IL17RD was also shown to interact with the elements of the canonical Wingless and Int (WNT) pathway, specifically β-catenin (CTNNB1) to modulate the metastatic and tumorigenic phenotype of breast cancer cells in vitro and in vivo [44]. Overexpression of IL17RD (SEF) restricted the transactivation of CTNNB1 to suppress the transcription of markers of epithelial-mesenchymal transition (EMT). The phenomenon of IL17RD regulation of EMT marker expression was consistently observed in normal breast epithelial cells stimulated with a combination of proinflammatory cytokines as well as in highly metastatic breast cancer cell lines [44]. It is possible that IL17RD functions as a docking site for phosphorylation of CTNN1 via an intermediate kinase to maintain its inactive state. Further studies are required in this regard.

### Other potential pathways

SEF (IL17RD) was shown to regulate Transforming Growth Factor beta (TGFβ) induced EMT response of epithelial cells [44, 45]; and has also been implicated in the pathophysiology of vascular remodeling diseases of the Bone Morphogenic Protein (BMP) pathway [46] suggesting that SEF might regulate the activation of the TGFβ/BMP pathway. Whilst the mechanism for SEF regulation of the above phenomena remains unknown, it is possible that TGFβ/BMP pathway by either acting as a scaffold to facilitate the interaction between endoglin and TGFβR/BMPR to fine tune the strength of signaling pathway, or by regulating the activation of the canonical Smad or the noncanonical ERK, p38 MAPK or phosphoinositide 3 kinase (PI3K/AKT) pathway. Additionally, the hSEF-S isoform was also shown to associate with and promote Lys63 polyubiquitination of TAK1 in HEK293T cells. In this context, SEF-S promoted apoptosis by activating both p38 MAPK and JNK pathways upon ultraviolet light exposure in an overexpression model [47].

### Sef (IL17RD) in diseases

Genetic alterations in *IL17RD* and dysregulation in its expression have been associated with a wide array of conditions, and are often accompanied by impairments in known IL17RD signaling partners.

#### Cancer

Expression of *IL17RD* has been shown to be inversely proportional to the degree of aggressiveness of tumors originating from the epithelia of organs such as breast, prostate, thyroid, ovaries and intestine [48–50]. In this context, ectopic expression of IL17RD was shown to regulate the biological response to FGF, EGF as well as the WNT signaling pathways by not only regulating the proliferation, migration and invasiveness of carcinoma cells, but also the expression of EMT markers to suppress the metastatic transformation of the tumors [35, 36, 44, 48, 49, 51, 52]. Additionally, IL17RD was shown to promote the sensitivity of resistant metastatic lung cancer cells to therapeutic MEK inhibitors [35]. SEF (IL17RD) has also been shown to regulate chromosomal segregation in colorectal cancer by suppressing RAS-mediated polyploidy via its known role as a modulator of cell cycle and apoptosis by suppressing the nuclear localization and phosphorylation of ERK [50]. The hSEF-b isoform was shown to suppress the angiogenic response in prostate cancer cells, suggesting a multimodal function for SEF as a tumor suppressor gene. [43]. Furthermore, mutations in *IL17RD* have also been associated with certain non-epithelial cancers, such as medullary thyroid carcinoma (MTC) [53] and follicular dendritic cell sarcoma (FDCS) [54] (see Table 1), in combination with perturbations in other RTK family members. Although the exact mechanism for dysregulation in IL17RD expression remains to be clarified, expression of *IL17RD* in carcinogenesis has been shown to be epigenetically regulated by proteins involved in DNA demethylation and repair such as AlkB homolog 3, alpha-ketoglutarate-dependent dioxygenase (ALKBH3) [55] and transcriptional repressors such as Zinc finger E-box-binding homeobox 1 (ZEB1) [35], as well as by microRNAs (miRs), such as miR200 [35].

**Table 1:** List of identified mutations in *IL17RD* occurring in diseased conditions

Disease association	Mutation	Type of mutation	Amino acid change	Inheritance	Zygosity	Citation
MTC	1385G > C	Missense	R462P			[53]
FDCS	T > C	Missense	I329T			[54]
KS	N/A	Missense	G35V		Heterozygous	[60]
KS	N/A	Missense	A221T		Heterozygous	[60]
KS, DP	392A > C*	Missense	K131T	de novo; AD	Heterozygous	[58, 61]
KS	485A > G	Missense	K162R	de novo	Heterozygous	[58]
nCHH, DP, KS	572C > T*	Missense	P191L	AD	Heterozygous	[56, 60, 61]
DP	N/A	Nonsense	W200X	AD	Heterozygous	[61]
KS	878C > T	Missense	P293L	AD	Heterozygous	[59]
KS	916C > T	Missense	P306S	de novo	Homozygous	[58]
KS	N/A	Missense	I329M		Heterozygous	[60]
KS	N/A	Missense	I329V		Heterozygous	[60]
KS	1136A > G	Missense	Y379C	AD	Heterozygous	[57, 58]
KS	1209G > A	Synonymous	G403G		Heterozygous	[58]
KS	1403C > T	Missense	S468L	AD	Heterozygous	[58]
nCHH	1592G > T	Missense	R531M		Heterozygous	[56]
nCHH	1608_1611del^#^	Frameshift	E536fs	AD	Heterozygous	[59]
nCHH	1697C > T^#^	Missense	P566L	AD	Homozygous	[59]
KS	1690 T > G	Missense	F564V		Heterozygous	[57]
KS	1730C > A	Missense	P577Q	AD	Homozygous	[58]
KS	2003C > T	Missense	S668F	AD	Heterozygous	[59]
KS	N/A*	Missense	S671L		Heterozygous	[60]
nCHH	2068 T > A	Missense	S690T		Heterozygous	[57]
KS	2204C > T	Missense	A735V	AD	Heterozygous	[58]
BAVM	676G > A	Missense	G226S	de novo	Heterozygous	[46]

^#^Mutations present on same individual *present in > 1 individual within the same study

#### Neuroendocrine diseases

High throughput sequencing and bioinformatics analyses [53] have implicated *IL17RD* as an important candidate in the ontogeny of syndromes associated with defects in the hypothalamic-pituitary–gonadal axis, predominantly Kallman syndrome (KS), as well as in disorders with overlapping pathophysiological parameters such as normosmic Congenital Hypogonadotropic Hypogonadism (nCHH) and delayed puberty (DP) [56–61]. Given its role as an important genetic contributor in the etiology of these disorders, *IL17RD* is used as an important genetic diagnostic marker in the pathophysiology of these diseases, with mutations occurring throughout all coding regions of the gene (see Table 1) [56–61]. However the genetic penetrance and phenotypic expressivity of symptoms was dependent on factors such as zygosity of the mutation as well as mutations occurring in other genetic loci, suggesting an oligogenic mode of action [58]. Although the accompanying mutations were often found in other members of the FGF synexpression group, and some of the identified mutations were shown to regulate IL17RD subcellular localization and FGF induced JNK activation in HEK293 cells [58] the exact functional role of *IL17RD* mutations in the etiology of these diseases requires further investigation. It is possible that IL17RD might affect initial Gonadotrophin Releasing Hormone (GnRH) secretory neuron specification and homeostatic capacity in the hypothalamus by virtue of its known function as a suppressor of neuronal differentiation, or that of those of its effector gonadotropic neurons in the pituitary [19, 31, 58]. IL17RD might also function downstream of gonadotropins to regulate the responsiveness of gonadal tissue and other affected neuronal networks (olfactory and auditory), given that IL17RD is expressed in olfactory placodes in mice [11, 58]; and dysregulation of IL17RD expression is known to affect the functioning of auditory circuits in zebrafish, chick and mice [12, 19, 28, 31, 58, 62, 63]. Alternately, given its oligogenic mode of interaction in the context of these diseases, it is possible that *IL17RD* might epistatically regulate the expression of another gene that might affect any of these functions. The diseases in which IL17RD mutations have been identified are summarized in Table 1.

#### Inflammatory and immunomodulatory diseases

Interactions between IL17RD and its associated ligands has been shown to have pathological relevance in different human and animal models of inflammatory diseases [26, 40]. *IL17RD* has been found to be expressed in synovial cells in patients suffering from rheumatoid arthritis [64], and its expression was found to be progressively downregulated in a manner depending on the severity of arthritic inflammation in mice as well as in humans [65]. Furthermore, studies from our lab have shown that IL17RD plays a crucial role in bone remodeling by regulating the balance between osteoblastogenesis and osteoclastogenesis via the ERK pathway in mice [66]. Collectively, this suggests that IL17RD might play a crucial role in regulating the inflammatory response in arthritic conditions by regulating the crosstalk between synovial cells and osteoclasts and consequent bone remodeling during arthritis.

IL17RD has also been shown to play a crucial role in fostering an immunoprotective response to systemic inflammation induced by TLR ligands in mice by regulating chemokine expression, suggesting that it may play an important role in regulating host response to pathogen exposure [21]. Concurrently, single nucleotide polymorphisms (SNPs) in *IL17RD* and the *TLR* family have been found to be associated with inflammatory phenotypes in malaria [67] (see Table 2). In another study utilizing a mouse model of in vivo cytokine induction, Mellett et al. showed that loss of *Il17rd* decreased peritoneal and pulmonary neutrophilia in response to IL17A through differential regulation of chemokine expression [26]. In a different cellular context, loss of *Il17rd* in keratinocytes was also shown to partially ameliorate imiquimod (IMQ) induced psoriasis by decreasing IL17A induced infiltration of neutrophils and γδ T cells into the epidermal layer of the skin and regulating keratinocyte expression of *Il23* [40]. This was accompanied by decreased expression of *IL17RD* in psoriatic skin as compared to normal tissue in humans [40, 68, 69]. However, another study identified an SNP within the intronic region of *IL17RD* in psoriasis [70], suggesting that alterations in either regulatory regions, posttranscriptional splicing, or modulation of transcript level via miRNAs might affect its mRNA stability in psoriasis, and consequently of *IL23*. Further, SNPs in *IL17RD* and *IL17RA* were also found to be associated with Crohn’s disease [71], however the exact mechanistic role of IL17RD in this context is still unknown. A list of SNPs that have been identified in *IL17RD* that are implicated in inflammatory disorders are summarized in Table 2.

**Table 2:** List of identified polymorphisms in *IL17RD* associated with diseased conditions

Disease	Annotation						Citation
Psoriasis	rs12495640						[71]
Malaria	rs6780995						[67]
Crohn's disease	rs768713						[70]
Crohn’s disease**	rs12495640	rs6788981	rs7374667				[70]
Crohn’s disease**	rs6809523	rs2129821	rs17057718	rs6780995	rs747089	rs6810042	[70]

**Inherited as a haplotype

#### Other diseases

Potentially pathogenic mutations in *IL17RD* have also been associated with non-syndromic cerebral arteriovenous malformations (BAVMs), along with mutations in members of RTK and BMP signaling superfamily [46]. Overexpression of IL17RD has also been shown to regulate the differentiation of ocular epithelial cells and of the surrounding lens fibers to regulate cataract formation [45, 72].

## Conclusions

Although there have been significant advancements in characterizing the IL17RD signaling pathways, interacting partners, and mechanisms of action, there are several issues that remain to be investigated, some of which have been mentioned previously. Because IL17RD interacts with multiple pathways, most of which have been investigated in vitro, further in vivo investigation using animal models and tissue specific gene targeting is required to shed new light on its role in these pathways and implications in disease. The mutations in *IL17RD* that have been shown to be associated with KS, nCHH and DP require further experimental characterization to define the functional consequences of these mutations. Moreover, determining whether IL17RD can be used as a biomarker for cancer, psoriasis or other diseases may have diagnostic and therapeutic implications. In this respect, characterizing the structural and conformational components of IL17RD involved in signaling through its various pathways may aid in identification of therapeutic targets for regulating dysfunctional IL17RD signaling.

## Data Availability

Not applicable.

## Abbreviations

AER: Apical ectodermal ridge
AR: Autosomal recessive
AD: Autosomal dominant
ALKBH3: AlkB homolog 3, alpha-ketoglutarate-dependent dioxygenase
BAVMs: Brain arteriovenous malformations
BMDM: Bone marrow derived macrophages
BMP: Bone morphogenic protein
DP: Delayed puberty
EGF: Epidermal growth factor
EGFR: EGF receptor
EMT: Epithelial-mesenchymal transition
ERK: Extracellular regulated kinase
FRS2α: FGFR substrate 2α
GnRH: Gonadotrophin releasing hormone
HEK293: Human embryonic kidney 293
IL: Interleukin
ILR: IL receptor
IMQ: Imiquimod
IκBα: Inhibitory subunit of NF Kappa B alpha
IKKβ: IκB kinase
IRAK: Interleukin 1 receptor associated kinase
JNK: C-Jun N-terminal kinase
KS: Kallman syndrome
Mal: MyD88 adaptor-like
MAPK: Mitogen activated protein kinase
MEF: Mouse embryonic fibroblasts
MEK: Mitogen activated protein kinase kinase
miR: MicroRNA
MIP: Macrophage inflammatory protein
MKK: Mitogen activated protein kinase kinase
MyD88: Myeloid differentiation primary response protein 88
nCHH: Normosmic congenital hypogonadatropic hypogonadism
NF-κB: Nuclear factor kappa light chain enhancer of activated B cells
NLS: Nuclear localization sequence
PDGF: Platelet derived growth factor
PDGFR: PDGF receptor
PI3K: Phosphoinositide 3 kinase
RTK: Receptor tyrosine Kinase
Sef: Similar expression to fgf
SEFIR: Sef/IL17R (SEFIR)
SH3: Src homology 3 (SH3)
SNP: Single nucleotide polymorphism
Spry: Sprouty
STIR: (SEFIR/TIR)
TAK: Transforming growth factor beta activated kinase
TGFβ: Transforming growth factor beta
Th17: T Helper 17
TIR: Toll/Interleukin-1 receptor
TNF: Tumor necrosis factor
TRAF: TNF receptor associated factor
TRIF: TIR-domain-containing adapter-inducing interferon β
TRAM: TRIF-related adaptor molecule
Wnt: Wingless and Int-1
ZEB1: Zinc finger E-box-binding homeobox 1
